# Conjunctival Swab Real Time-PCR in *Leishmania infantum* Seropositive Dogs: Diagnostic and Prognostic Values

**DOI:** 10.3390/biology11020184

**Published:** 2022-01-24

**Authors:** Maria Alfonsa Cavalera, Andrea Zatelli, Rossella Donghia, Jairo Alfonso Mendoza-Roldan, Floriana Gernone, Domenico Otranto, Roberta Iatta

**Affiliations:** 1 Department of Veterinary Medicine, University of Bari, 70010 Bari, Italy; mariaalfonsa.cavalera@uniba.it (M.A.C.); jairo.mendozaroldan@uniba.it (J.A.M.-R.); floriana.gernone@uniba.it (F.G.); domenico.otranto@uniba.it (D.O.); 2 Unit of Research Methodology and Data Sciences for Population Health, “Salus in Apulia Study” National Institute of Gastroenterology “S. de Bellis” Research Hospital, 70013 Bari, Italy; rossydonghia@gmail.com; 3 Faculty of Veterinary Sciences, Bu-Ali Sina University, Hamedan 65175-4161, Iran; 4 Interdisciplinary Department of Medicine, University of Bari, 70124 Bari, Italy; roberta.iatta@uniba.it

**Keywords:** canine leishmaniosis, conjunctival swab, diagnostic tests, dog, IFAT, *Leishmania*, PCR

## Abstract

**Simple Summary:**

This 10-month-long study describes how conjunctival swab (CS) real time-PCR (*q*PCR) failed to be a useful diagnostic and prognostic test for canine leishmaniosis (CanL). From the beginning of the study (October 2020), a limited number of *Leishmania infantum* seropositive dogs remained CS *q*PCR positive in August 2021, and none of them developed an active CanL. Therefore, the detection of *L. infantum* kDNA by *q*PCR, even highly sensitive, may be considered “a random” if not accompanied by a significant clinical score for CanL and/or other direct diagnostic tests positivity. Hence, in seropositive dogs with CS *q*PCR positivity, sampling time and season variability of results should be considered. In this scenario, testing other significant biological samples (e.g., lymph node, bone marrow, and spleen), although invasive, is strongly advised.

**Abstract:**

Conjunctival swabs (CS) are the most promising non-invasive samples for the diagnosis and the regular screening of *Leishmania infantum* infection in dogs although knowledge on their diagnostic performance is still inconclusive. This study evaluates CS real time-PCR (*q*PCR) analysis for the diagnosis of canine leishmaniosis (CanL) and its prognostic value in seropositive dogs from an endemic area. In October 2020 (T0), 26 dogs were enrolled, divided in two groups according to anti-*L. infantum* antibody titres (*n* = 13, group low titre (LT) and *n* = 13, group high titre (HT)), and followed-up in August 2021. At both timepoints, animals underwent clinical examination, complete blood count and biochemical analyses, and serological (indirect fluorescent antibody test) and molecular (CS and peripheral blood *q*PCR) testing. At T0, 10 out of 26 enrolled dogs were positive at CS *q*PCR, with the number of positive animals significantly higher in group HT than in LT. After 10 months, only 5 out of 21 dogs that completed the trial still tested CS *q*PCR positive, and none of them developed an active CanL based on clinical score and antibody titre. None of the dogs required any leishmanicidal and/or leishmaniostatic treatments. This prospective study showed unsatisfying diagnostic and prognostic performances of CS *q*PCR analysis in *L. infantum* seropositive asymptomatic dogs from an endemic area.

## 1. Introduction

Zoonotic canine leishmaniosis (CanL) by *Leishmania infantum* transmitted by sand flies represents a threat for the health of dogs, the principal reservoir hosts for this protozoan [1]. A correct diagnostic process is crucial to identify both dogs with clinical signs compatible with *L. infantum* infection and asymptomatic carriers, which represent the largest infected population (up to 85%) in endemic geographical areas [2,3,4]. Etiological diagnosis of CanL mainly relies on serological and parasitological methods. In veterinary practice and clinical and epidemiological studies on *L. infantum*, quantitative serological tests, such as indirect fluorescent antibody test (IFAT) and enzyme linked immunosorbent assays (ELISA), have been largely used due to their high diagnostic performance with a sensitivity and specificity close to 100% in dogs with clinical signs [5]. Indeed, anti-*L. infantum* antibody quantification is a useful tool for screening large number of samples and quantifying the exposure to *L. infantum* of canine population. Furthermore, quantitative serodiagnosis is used to confirm clinical suspicion since dogs with high mean clinical scores usually display high levels of antibodies [6]. The significant correlation between the severity of the disease and the level of antibody titres [6,7,8] also applies to the concentration of circulating immune complexes (CICs), which have been recognized to play a pathogenic role in sick dogs [9,10,11]. Recently, a seasonal variation in canine anti-*L. infantum* antibody titres has been described and should be considered in the interpretation of annual antibody screening test results and to make clinical decisions about staging, treatment, and prevention of CanL in dogs [12]. Parasitological techniques, including cytology, histology, immunohistochemistry, parasite culture in an appropriate medium, and xenodiagnosis, can unveil *L. infantum* infection even though the final diagnosis of an active CanL should rely on clinical findings and clinicopathologic tests [13]. Fine-needle aspiration cytology (FNAC) from mucocutaneous lesions or enlarged lymph node in dogs with clinical signs and/or laboratory abnormalities potentially consistent with CanL is an easy, cheap, and reliable diagnostic method to demonstrate the presence of *L. infantum* amastigotes [14]. Conversely, it should be noted that lymphoid tissue material could be difficult to obtain in sufficient amount when palpable nodes are not enlarged [15] and that lymph node enlargement is a not always evident clinical sign, becoming appreciable only several months after *L. infantum* infection [13,16]. Bone marrow FNAC is considered as one of the most sensitive techniques for a reliable diagnosis of CanL even though it is not a complication-free procedure, as it can cause pain, haemorrhage, and infection [14]. Compared with FANC, histology can provide the presence of *L. infantum* along with additional information on the cytoarchitectural pattern of the lesions although it is more expensive and time-consuming, and the identification of amastigotes may be more difficult than in cytologic samples [14]. Parasite culture and xenodiagnosis are difficult to apply for routine practice because they result unpractical and generally restricted to specialized reference centers [4]. Since the 90s [17], the use of molecular tests has represented a major step forward toward increasing diagnostic testing for CanL. Conventional, nested, and real-time polymerase chain reactions (PCR) are sensitive and specific methods for the detection of *Leishmania* spp. infection both in clinically suspected and apparently healthy dogs, with the latter group being a potential source of the parasite to the phlebotomine vectors [4,18,19]. The diagnostic sensitivity of molecular assays relies considerably on the type of tissue evaluated, namely bone marrow, lymph node, and spleen as the most suitable samples for detecting *Leishmania* DNA [14,18], and on the PCR target being the kinetoplast DNA minicircle (kDNA) the most sensitive [20]. Recently, much attention has been paid to the use of samples for molecular diagnosis collected with non-invasive techniques, such as conjunctival, oral, vulvar, or nasal swabs [20]. In particular, conjunctival swabs (CS) appear to be the most promising non-invasive sample for the regular screening of the canine population [21,22] and for the diagnosis in dogs with and without clinical signs compatible with CanL [21,22,23,24,25,26,27], as it is potentially able to provide positive results earlier than other tissues [22]. Furthermore, CS are listed between the first-choice samples for PCR in the majority of guidelines for CanL [4,28]. However, knowledge on the diagnostic performance of this non-invasive sampling method is still inconclusive due to the variability in the data collected from previous trials (e.g., diagnostic value in symptomatic and/or asymptomatic dogs, PCR protocols, sampling techniques) [23,26,27,29] and a few pieces of information available from longitudinal studies [30,31]. Therefore, the present study aims to assess the diagnostic performance of CS real time-PCR (*q*PCR) for the detection of *L. infantum* in IFAT seropositive dogs from a CanL endemic area. The prognostic value of CS *q*PCR has been also evaluated.

## 2. Materials and Methods

### 2.1. Dog Population, Sampling, and Follow-Up

This 10-month-long study was approved by the ethical committee of the Department of Veterinary Medicine, University of Bari, Italy (Approval number, Prot. Uniba 24/2020). In October 2020, *L. infantum*-seropositive dogs from a shelter located in a CanL endemic area in Apulia region, southern Italy (40.419326° N, 18.165582° E, Lecce), underwent a complete physical examination, and a clinical score ranging from 0 to 19 was assigned (modified from [32]) (Table 1). From each dog, peripheral blood (PB) (5 mL divided in a tube with EDTA (2 mL) and a tube with serum separator gel (3 mL)) was sampled. Exfoliative epithelial cells were collected from the right and left conjunctiva using a sterile cotton swab intended for bacteriological isolation. Samples were transported to the laboratory within 4 h from collection, where each PB sample with EDTA was divided into two aliquots of 1 mL each (i.e., for a complete blood count (CBC) analysis and for molecular testing as described below). Serum was obtained from blood sample in the serum separator tube by centrifugation (1500 g for 15 min) and divided into two aliquots for biochemical analysis and serological testing. Enrolled dogs were divided in two groups according to the anti-*L. infantum* antibody titres (i.e., ≤1:320 (group low titre, LT) and >1:320 (group high titre, HT)) [4] and followed-up in August 2021 when all animals underwent repeated clinical examination, CBC, biochemical analysis, and serological and molecular testing.

### 2.2. Serological Testing

Serum samples were tested for anti-*L. infantum* IgG by IFAT as previously described [3]. Samples were considered positive if there was clear cytoplasmic and membrane fluorescence of *L. infantum* promastigotes from a cut-off dilution of 1:80. Positive sera were titrated by serial dilutions (i.e., up to 1:2560) until negative results were obtained. All serological tests were read in a double-masked manner by two different operators.

### 2.3. Molecular Testing

PB samples and CS were subjected to DNA extraction using the DNeasy Blood and Tissue Extraction Kit (Qiagen) respectively, according to manufacturer’s instructions. Detection of 120 base pair fragment of *L. infantum* kinetoplast DNA minicircle was achieved by real time-PCR (*q*PCR) using primers, probes, and protocol as previously described [33]. Samples were scored as positive when a threshold cycle up to 37 was recorded.

### 2.4. Statistical Analyses

Dogs’ characteristics are reported as mean ± standard deviation (M ± SD) and as frequencies and percentages (%) for categorical. For testing the associations between two independent groups (i.e., LT and HT), the Chi-square test or Fisher’s exact test for categorical variables was used, when necessary, while the Wilcoxon rank sum (Mann–Whitney) test was used for continuous variables. The test of equality for matched data was used to compare the difference between pairs of observation in the groups in time (October 2020 and August 2021) for continuous variables, while McNemar’s test or McNemar–Bowker test for categorical variables were used. The Spearman rank correlation coefficient was used to test the strength and direction of association exists between two variables examined (i.e., between IFAT, PB *q*PCR, CS *q*PCR, and clinical score). When testing the null hypothesis of no association, the probability level of error at two tails was 0.05. If a dog dropped out, the intention-to-treat (ITT) analysis was followed using the last molecular result assessed as the final result. All the statistical computations were made using StataCorp. 2021. Stata Statistical Software: Release 17. College Station, TX: StataCorp LLC.

## 3. Results

Twenty-six neutered *L. infantum*-seropositive dogs (*n* = 12 males, *n* = 14 females) of different ages (7.8 ± 3.05 years) and breeds (*n* = 24 mixed-breed, *n* = 1 German shepherd, and *n* = 1 Pointer) were included in the study, divided in two groups of 13 animals each according to anti-*L. infantum* antibody titres (i.e., group LT and group HT), and followed-up after 10 months as shown in Table 2.

In August 2021, five enrolled dogs were lost to follow-up due to death not related to CanL (#4LT, #5LT, #13HT) or adoption (#1LT, #12HT) (Table 2). At the beginning of the study, 10 out of 26 enrolled dogs (38.46%) were positive at CS *q*PCR (i.e., *n* = 2 (15,38%) in group LT and *n* = 8 (61.54%) in group HT) (Table 2 and Table 3).

The number of animals with positive CS *q*PCR was significantly higher in group HT than in group LT (Table 3). No statistically significant difference was found between group LT and group HT in PB *q*PCR results and clinical score (Table 3). After 10 months, out of 21 dogs that completed the trial (i.e., *n* = 10 in group LT, *n* = 11 in group HT), five animals that tested CS *q*PCR positive in October 2020 (i.e., *n* = 2 in group LT and *n* = 3 in group HT) were still positive in August 2021. Three dogs of group HT (#2HT, #3HT, #7HT) became negative at CS *q*PCR and reduced the anti-*L. infantum* antibody titres during the study period. One (#1LT) and two (#5HT, #6HT) dogs of group LT and HT, respectively, were found positive at CS *q*PCR in August 2021 (Table 2). No statistically significant difference was detected in molecular results (i.e., CS and PB *q*PCR) and clinical scores between October 2020 and August 2021 considering either all the animals enrolled in the study (Table 4) or dogs in the groups LT and HT (Table 5).

A strong positive correlation between IFAT, PB, and CS *q*PCR in October 2020 and between clinical score and PB *q*PCR in October 2020 and August 2021 was found (Table 6). During the study period, all animals had a clinical score < 3 out of 19 (modified from [32]) (Table 2) and hematological and biochemical parameters mainly within normal limits; they did not require leishmanicidal nor leishmaniostatic treatments, and their diet was not changed.

## 4. Discussion

This prospective study showed unsatisfying diagnostic and prognostic performances of CS *q*PCR analysis in *L. infantum* seropositive asymptomatic dogs from a CanL endemic area in southern Italy. Though the CS is currently described as a promising non-invasive sample for providing consistent diagnosis or performing epidemiological surveys of CanL through conventional PCR [21,27] and *q*PCR [26,28,34,35] in both symptomatic [22,23,24] and asymptomatic dogs [21], our results were highly variable. This study shows a lower CS *q*PCR positivity (i.e., up to 38.5%) if compared with other studies in *L. infantum* seropositive symptomatic and asymptomatic dogs [22,25,26,36]. Numerous studies reported a significant correlation between the level of antibody titres and the severity of the disease [6,7,8] as well as the CICs concentration, which have a widely recognized pathogenic role in naturally infected dogs [9,10,11]. Even if in October 2020, the number of animals with positive CS *q*PCR was significantly higher in group HT than in group LT, a limited number of animals remained CS *q*PCR positive in August 2021 (i.e., *n* = 2 in group LT and *n* = 3 in group HT), and none of them developed an active CanL based on clinical score and antibody titre (Table 2), demonstrating a poor prognostic value of this analysis. It can be hypothesized that dogs with higher anti-*L. infantum* antibody titres in October (i.e., end of the sand fly season in the study area [37,38]) have been exposed to infected sand fly bites for a longer period, being more likely to have an up-regulated humoral immune response [12] and they have a higher probability to identify the parasite’s DNA in the conjunctiva closed to hairless periocular region (e.g., eyelids are common site of sand fly bites) [39]. Moreover, the significant difference in the number of animals with positive CS *q*PCR between the group HT and LT was not found in August 2021, demonstrating an extreme variability in the results, likely related to the sampling period (i.e., sand fly seasonality and transmission time). Besides, anti-*L. infantum* antibody titres also mainly decreased in August 2021, with three out of 21 dogs becoming negative, showing a sand fly season-related serological variability as previously demonstrated within [40] and between transmission seasons [41] as well as between transmission and non-transmission seasons [12]. Therefore, the detection of *L. infantum* kDNA by *q*PCR, even highly sensitive, can be considered “a random” if not accompanied by a significant clinical score for CanL and/or other direct diagnostic tests positivity detected on biological samples, such as lymph node and/or bone marrow [26]. Interestingly, three dogs of group HT became negative at CS *q*PCR in August 2021. These negative CS *q*PCR results can be related to the low parasitic load at the collection site due to an adequate immune response controlling the spread of pathogen in tissues, such as mucous membranes and skin [9], and/or for serological cross-reactivity. Indeed, the co-infection with other trypanosomatids may induce seroconversion/increased antibody titre detected by serological testing in dogs resulting negative by *L. infantum q*PCR [42,43]. Recently, dogs were firstly found seropositive for *L. tarentolae* and *L. infantum* by IFAT in the same area of the present study [38], therefore suggesting a possible serological cross-reaction. *Leishmania infantum* DNA has been detected in PB samples of only a few seropositive dogs (*n* = 4 in group HT in October 2020 and *n* = 1 in group HT in August 2021), confirming that PB is not the ideal tissue for molecular diagnosis of CanL [19]. However, a strong positive correlation between PB *q*PCR and clinical score was found in both time points (Table 6), suggesting that PB *q*PCR may be a useful test for high positive predictive value based on clinical evaluation. The potential limitations of the present study are the small number of dogs enrolled from the same study area even though animals’ loss to follow-up (dead or adopted) is one of the most frequent occurrences in a kennel, and to avoid this bias, the ITT analysis was applied. Furthermore, the shelter environment offers the possibility of studying a homogenous canine population over long periods of time and under the same living conditions. Moreover, factors related to the sampling technique and the molecular test performed (e.g., PCR method, target gene) may have influenced the obtained results. Nevertheless, it should be considered that *q*PCR targeting the parasite kDNA herein used represents to date one of the most sensitive methods for the molecular detection of *L. infantum* [19]. Finally, future studies involving the sampling and molecular analysis of CS at different time points during the transmission (e.g., beginning, middle, and end of sand fly season) as well as in the non-transmission periods are needed to support the hypothesis that CS *q*PCR results may be highly influenced by exposure time of dogs to *L. infantum* infected sand flies.

## 5. Conclusions

This 10-month-long study describes how CS *q*PCR failed to be a useful diagnostic and prognostic test for CanL. Hence, in seropositive dogs with CS *q*PCR positivity, sampling time and season variability should be considered. In this scenario, testing other significant biological samples (e.g., lymph node, bone marrow, and spleen), although invasive, is strongly advised.

## Figures and Tables

**Table 1:** Clinical sign-based score for canine leishmaniosis ranging between 0 and 19 (modified from [32]).

Systemic signs	Attitudes	active	0
		apathetic	1
	Ectoparasites	absence	0
		fleas	1
		fleas and ticks	2
	Body condition score	3–5/5	0
		2/5	1
		1/5	2
	Lymph node	normal	0
		enlarged	1
	Mucosa colour	normal	0
		pale	1
	Bleeding	absence	0
		presence	1
Cutaneous signs	Bristles	good	0
		regular	1
		bad/opaque	2
	Muzzle/Ear lesions	absence	0
		presence	1
	Nails	normal	0
		long/onychogryphosis	1
	Skin lesion	absence	0
		presence	1
		ulcer	2
	Muzzle depigmentation	absence	0
		presence	1
	Alopecia	absence	0
		presence	1
Ocular signs	Blepharitis	absence	0
		presence	1
	Keratoconjunctivitis	absence	0
		serous	1
		mucopurulent	2

**Table 2:** Enrolled dogs divided in two groups according to the anti-*L. infantum* antibody titres (i.e., ≤1:320 (group low titre, LT) and >1:320 (group high titre, HT)) and followed-up in August 2021.

Group	Dog #	October 2020				August 2021			
		*L. infantum* IFAT	PB *q*PCR (Ct Value)	CS *q*PCR (Ct Value)	Clinical Score (0–19)	*L. infantum* IFAT	PB *q*PCR (Ct Value)	CS *q*PCR (Ct Value)	Clinical Score (0–19)
Group LT	1 LT °	1:80	neg	neg	0	nd	nd	nd	nd
	2 LT	1:80	neg	neg	0	neg	neg	neg	0
	3 LT	1:160	neg	neg	0	neg	neg	neg	0
	4 LT °°	1:160	neg	neg	0	nd	nd	nd	nd
	5 LT °°	1:160	neg	neg	0	nd	nd	nd	nd
	6 LT	1:320	neg	neg	0	neg	neg	neg	0
	7 LT	1:320	neg	pos (37)	0	1:80	neg	pos (36)	0
	8 LT	1:320	neg	neg	0	1:320	neg	neg	0
	9 LT	1:320	neg	neg	0	1:160	neg	neg	0
	10 LT	1:320	neg	pos (36)	0	1:80	neg	pos (32)	0
	11 LT	1:320	neg	neg	0	1:80	neg	pos (32)	0
	12 LT	1:320	neg	neg	0	1:160	neg	neg	0
	13 LT	1:320	neg	neg	0	1:160	neg	neg	0
Group HT	1 HT	1:640	neg	pos (37)	0	1:640	neg	pos (31)	0
	2 HT	1:640	neg	pos (33)	0	1:320	neg	neg	0
	3 HT	1:640	neg	pos (32)	0	1:320	neg	neg	1
	4 HT	1:640	neg	neg	0	1:320	neg	neg	0
	5 HT	1:640	neg	neg	0	1:160	neg	pos (30)	0
	6 HT	1:640	neg	neg	0	1:320	neg	pos (35)	0
	7 HT	1:640	neg	pos (36)	0	1:320	neg	neg	0
	8 HT	1:1280	neg	neg	0	1:640	neg	neg	0
	9 HT	1:1280	pos (29)	pos (31)	1	1:320	pos (31)	pos (26)	1
	10 HT	1:2560	neg	neg	0	1:640	neg	neg	0
	11 HT	1:2560	pos (27)	pos (26)	1	1:1280	neg	pos (28)	2
	12 HT °	1:2560	pos (32)	pos (28)	0	nd	nd	nd	nd
	13 HT °°	1:2560	pos (27)	pos (23)	0	nd	nd	nd	nd

Abbreviations: IFAT, Indirect Fluorescent Antibody Test; PB, peripheral blood; *q*PCR, Real Time-PCR; CS, conjunctival swabs; nd, not determined. # dog identification code; ° adopted dog; °° dead dog.

**Table 3:** Comparison of diagnostic tests results and clinical score between dogs of the low titre group (Group LT) and the high titre group (Group HT) in October 2020 and in August 2021.

Parameters	October 2020			August 2021		
	Group LT (*n =* 13)	Group HT (*n =* 13)	*p* ^ψ^	Group LT (*n =* 10)	Group HT (*n =* 11)	*p* ^ψ^
IFAT			**<0.001**			**0.004**
0	0 (0.00)	0 (0.00)		3 (30.00)	0 (0.00)	
1:80	2 (15.38)	0 (0.00)		3 (30.00)	0 (0.00)	
1:160	3 (23.08)	0 (0.00)		3 (30.00)	1 (9.09)	
1:320	8 (61.54)	0 (0.00)		1 (10.00)	6 (54.55)	
1:640	0 (0.00)	7 (53.85)		0 (0.00)	3 (27.27)	
1:1280	0 (0.00)	2 (15.38)		0 (0.00)	1 (9.09)	
1:2560	0 (0.00)	4 (30.77)		0 (0.00)	0 (0.00)	
CS *q*PCR			**0.04**			0.69 ^§^
Negative (−)	11 (84.62)	5 (38.46)		7 (53.85)	6 * (46.15)	
Positive (+)	2 (15.38)	8 (61.54)		6 (46.15)	7 * (53.85)	
PB *q*PCR			0.10			0.99
Negative (−)	13 (100.00)	9 (69.23)		10 (76.92)	10 * (76.92)	
Positive (+)	0 (0.00)	4 (30.77)		3 (23.08)	3 * (23.08)	
Clinical Score			0.48			0.34
0	13 (100.00)	11 (84.62)		10 (100.00)	8 (72.73)	
1	0 (0.00)	2 (15.38)		0 (0.00)	2 (18.18)	
2	0 (0.00)	0 (0.00)		0 (0.00)	1 (9.09)	

Abbreviations: IFAT, Indirect Fluorescent Antibody Test; PB, peripheral blood; *q*PCR, Real Time-PCR; CS, conjunctival swabs. ^ψ^ Fisher’s test or ^§^ Chi-square test when necessary; * the intention-to-treat analysis was applied. Values in bold indicate statistically significant results.

**Table 4:** Comparison between October 2020 and August 2021 in dogs enrolled in the study.

Parameters	October 2020 (*n* = 26)	August 2021 (*n* = 21)	*p* ^ψ^
IFAT			0.42 ^¥^
0	0 (0.00)	3 (14.29)	
1:80	2 (7.69)	3 (14.29)	
1:160	3 (11.54)	4 (19.05)	
1:320	8 (30.77)	7 (33.33)	
1:640	7 (26.92)	3 (14.29)	
1:1280	2 (7.69)	1 (4.76)	
1:2560	4 (15.38)	0 (0.00)	
CS *q*PCR			0.51
Negative (−)	16 (61.54)	13 * (50.00)	
Positive (+)	10 (38.46)	13 * (50.00)	
PB *q*PCR			0.62
Negative (−)	22 (84.62)	20 * (76.92)	
Positive (+)	4 (15.38)	6 * (23.08)	
Clinical Score			0.37 ^¥^
0	24 (92.31)	18 (85.71)	
1	2 (7.69)	2 (9.52)	
2	0 (0.00)	1 (4.76)	

Abbreviations: IFAT, Indirect Fluorescent Antibody Test; PB, peripheral blood; *q*PCR, Real Time-PCR; CS, conjunctival swabs. ^ψ^ McNemar’s test; ^¥^ McNemar–Bowker test; * the intention-to-treat analysis was applied.

**Table 5:** Comparison of diagnostic tests results and clinical score between October 2020 and August 2021 in dogs of the low titre (LT) and high titre (HT) groups.

Parameters	Group LT			Group HT		
	October 2020 (*n =* 13)	August 2021 (*n =* 10)	*p* ^ψ^	October 2020 (*n =* 13)	August 2021 (*n =* 11)	*p* ^ψ^
IFAT			0.19 ^¥^			0.57 ^¥^
0	0 (0.00)	3 (30.00)		0 (0.00)	0 (0.00)	
1:80	2 (15.38)	3 (30.00)		0 (0.00)	0 (0.00)	
1:160	3 (23.08)	3 (30.00)		0 (0.00)	1 (9.09)	
1:320	8 (61.54)	1 (10.00)		0 (0.00)	6 (54.55)	
1:640	0 (0.00)	0 (0.00)		7 (53.85)	3 (27.27)	
1:1280	0 (0.00)	0 (0.00)		2 (15.38)	1 (9.09)	
1:2560	0 (0.00)	0 (0.00)		4 (30.77)	0 (0.00)	
PB *q*PCR			0.08			0.99
Negative (−)	13 (100.00)	10 (76.92)		9 (69.23)	10 * (76.92)	
Positive (+)	0 (0.00)	3 (23.08)		4 (30.77)	3 * (23.08)	
CS *q*PCR			**0.04**			0.99
Negative (−)	11 (84.62)	7 (53.85)		5 (38.46)	6 * (46.15)	
Positive (+)	2 (15.38)	6 (46.15)		8 (61.54)	7 * (53.85)	
Clinical Score			^--^			0.37 ^¥^
0	13 (100.00)	10 (100.00)		11 (84.62)	8 (72.73)	
1	0 (0.00)	0 (0.00)		2 (15.38)	2 (18.18)	
2	0 (0.00)	0 (0.00)		0 (0.00)	1 (9.09)	

Abbreviations: IFAT, Indirect Fluorescent Antibody Test; PB, peripheral blood; *q*PCR, Real Time-PCR; CS, conjunctival swabs. ^ψ^ McNemar’s test; ^¥^ McNemar–Bowker test; * the intention-to-treat analysis was applied. Values in bold indicate statistically significant results.

**Table 6:** Correlation matrix based on Spearman rank correlation coefficient (ρ) between indirect fluorescent antibody test (IFAT), peripheral blood (PB) real time-PCR (*q*PCR), CS *q*PCR, and Clinical Score during October 2020 and August 2021.

**October 2020**				
**ρ ^¥^**	**IFAT**	**PB *q*PCR**	**CS *q*PCR**	**Clinical Score**
IFAT	--	--	--	--
PB *q*PCR	0.59 (**0.001**)	--	--	--
CS *q*PCR	0.38 (**0.05**)	0.33 (0.10)	--	--
Clinical Score	0.37 (0.06)	0.66 (**0.0003**)	0.24 (0.23)	--
**August 2021**				
**ρ ^¥^**	**IFAT**	**PB *q*PCR**	**CS *q*PCR**	**Clinical Score**
IFAT	--	--	--	--
PB *q*PCR	0.11 (0.62)	--	--	--
CS *q*PCR	−0.04 (0.87)	0.13 (0.58)	--	--
Clinical Score	0.38 (0.09)	0.51 (**0.02**)	0.09 (0.70)	--

^¥^ ρ, Spearman’s Rho. Values in bold indicate statistically significant results.

## Data Availability

The datasets generated and/or analysed during the current study are available from the corresponding author on reasonable request.
